# Implementation of maternal death audits and changes in maternal health care in Cambodia, 2010–2017

**DOI:** 10.5365/wpsar.2024.15.4.1127

**Published:** 2024-11-18

**Authors:** Rathavy Tung, Rattana Kim, Matthews Mathai, Kannitha Cheang, Howard L Sobel

**Affiliations:** a Department of Maternal and Child Health, Ministry of Health, Phnom Penh, Cambodia.; b World Health Organization Regional Office for the Western Pacific, Manila, Philippines.; c World Health Organization Representative Office for Cambodia, Phnom Penh, Cambodia.

## Abstract

**Objective:**

Cambodia is one of seven countries globally that met Millennium Development Goal 5A: reduction of maternal deaths by at least 75% between 1990 and 2015. The maternal death audit (MDA) was instituted in 2004 to support the improvement of maternal care. We evaluated progress in MDA implementation and maternal health services in Cambodia between 2010 and 2017.

**Methods:**

International experts and the national MDA committee members assessed all case abstracts, investigation questionnaires and audit meeting minutes covering all maternal deaths reported in Cambodia in 2010 and 2017 for quality of classification, data, care and recommendations. They convened provincial MDA committees to conduct similar assessments and develop evidence-based recommendations. Differences in data from the two years were assessed for significance using χ^2^ and Fisher’s exact tests.

**Results:**

In 2010 and 2017, 176 and 59 maternal death cases were reported, respectively. Cases were more likely in 2017 than in 2010 to have antenatal care (90.0% vs 68.2%, *P* = 0.004), give birth in a facility (81.6% vs 55.3%, *P* = 0.01) and receive a prophylactic uterotonic (95.7% vs 73%, *P* < 0.02) for postpartum haemorrhage and magnesium sulfate (66.7% vs 37%, *P* = 0.18) for preeclampsia/eclampsia. However, additional interventions and improved timeliness of referral with equipped and competent staff were identified as critical. Data quality prevented the classification of one fourth of cases during both periods. The quality of MDA recommendations improved from 2.8% in 2011 to 42% in 2018.

**Discussion:**

Improvements in maternal care are reflected in the increased antenatal care, facility births and better postpartum haemorrhage and preeclampsia/eclampsia management. However, additional care management improvements are needed. The MDA reporting needs to improve data completeness and make more specific recommendations to address causes of death.

Pregnant women are among the most vulnerable populations, and this increases during public health emergencies whether from disasters or infectious disease outbreaks. The Asia Pacific Health Security Action Framework (*1*) notes the critical role that resilient health systems play in delivering equitable and timely health services before, during and after a public health emergency, especially for vulnerable populations. It further recognizes the mutually reinforcing role of health security and health systems. The resilience of health systems to deliver services during events is highly associated with how strong the system was before the event. Thus, priority needs to focus on continuity planning to maintain obstetric and other essential health services during emergencies and strengthen routine care and surveillance. Maternal death surveillance and response systems provide critical information for strengthening routine maternal health-service delivery systems to mitigate pregnancy-related risks, including those from public health emergencies.

Cambodia is one of a few countries that met the target of Millennium Development Goal (MDG) 5A: reduction of maternal deaths by at least 75% between 1990 and 2015. (*2*) In this context, Cambodia’s Ministry of Health issued the Fast-track Initiative Roadmap for Reducing Maternal and Newborn Mortality (2010–2015 and 2016–2020), (*3*, *4*) which recommended skilled attendance at birth and birth spacing. The government achieved this by: (1) recruiting midwives for all health centres; (2) enabling them to provide basic emergency obstetric care, family planning counselling and implement safe abortion care through direct training, supportive supervision and skills building during periodic refresher skills training; (3) providing financial incentives to midwifery teams for each facility birth that ended with a live mother and newborn; (4) removing financial barriers by establishing the Health Equity Fund (initiated in 2008) (*5*) and other financial schemes; and (5) developing behavioural change communication.

In Cambodia, maternal deaths were estimated to have reduced from 1020 to 161 per 100 000 live births between 1990 and 2015, while births attended by skilled personnel and the number of women reporting at least four antenatal care visits tripled between 2000 and 2014. (*6*-*8*)

The World Health Organization (WHO) recommends that countries conducting maternal death surveillance and response (MDSR) address critical moments where a woman’s life could have been saved. (*9*, *10*) Case studies found that while MDSR is well accepted, its integration into health systems is limited. (*11*) Evaluations in Zimbabwe found that inadequate health-worker knowledge and the scarcity of guidelines and notification forms impeded MDSR functionality. (*12*) Studies in Nigeria, Rwanda, United Republic of Tanzania and Zimbabwe found that 55% of facilities could demonstrate improvements in “evidence of routine integration” of the maternal and perinatal death surveillance and response (MPDSR) process, but few facilities had mechanisms to promote a no-blame environment. (*13*) In Ethiopia, the triangulation of MDSR and emergency obstetric care data identified postpartum haemorrhage (PPH) (10–27%) and severe preeclampsia/eclampsia (10–24%) as common immediate causes of death, with delayed arrival at a health facility and transfer to an appropriate level of care accounting for 32–40% and 22–29% of underlying factors, respectively. Half (48%) of women who died of PPH received uterotonics, while 72% who died of severe preeclampsia/eclampsia received anticonvulsants. (*14*)

This report aims to highlight progress noted during two internationally led peer review assessments of the Maternal Death Audit (MDA) implementation and maternal health-service quality conducted in 2010 and 2017 in Cambodia.

## Methods

### Routine maternal death audits

Cambodia instituted the National Protocol on Maternal Death Audit, including confidential enquiry and verbal autopsies, in 2004. (*15*) The protocol included: the MDA steps; organizational structure and terms of reference for members; standardized templates, including notification and investigation forms to be completed for each case and a narrative form to briefly describe the events from onset until death; and a table of recommendations from audit meetings. The protocol states that maternal deaths should be reported as soon as possible, investigated within 2 weeks and reviewed at provincial MDA committee audit meetings within a week thereafter. During reviews, gaps and contributing factors are identified and recommendations made to prevent future deaths. These are distributed to provincial health departments and operational districts and assessed for implementation at subsequent audit meetings. (*15*)

### International expert-facilitated review

International experts facilitated the review of all reported maternal deaths from 2010 in a 2011 review workshop. This led to revisions to the national MDA protocol (2014), (*16*) including: the revision of the national and provincial MDA committees’ structure and terms of reference; the establishment of a regular meeting for the national MDA committee to monitor and provide technical support to all provincial MDA committees; and the strengthening of the recommendations and response portions of the protocol. A similar review workshop was conducted in 2018 to assess all reported maternal deaths in 2017.

Prior to the workshop, international experts and the national MDA committee assessed all maternal death case abstracts, investigation data collection forms, and minutes of audit meetings from each of the 24 provinces and Phnom Penh (the capital region) in 2010 and 2017. They classified cases as maternal deaths (direct or indirect), fortuitous deaths (those that occur due to causes unrelated to pregnancy), not a maternal death (the woman was neither pregnant nor had she been pregnant in the preceding 42 days) or unclassifiable (maternal death of unknown cause). They then identified the cause, timing and place of death and assessed whether provincial MDA committee recommendations addressed the key moments in which a woman’s life could have been saved, including recognition of underlying problems within the health system, such as the lack of essential medicines and health-care workers with the capacity to manage obstetric emergencies. They commented on data quality, including data needed to improve case classification and recommendations. Finally, they compared the reported numbers of maternal deaths across the country with the United Nations maternal death estimates for Cambodia.

On 2–3 June 2011 and 14–16 November 2018, the national MDA committee convened MDA assessment workshops with three members from each subnational MDA committee to review MDA reports and committee recommendations. Participants were divided into eight groups of nine people from three provinces (three per province). Each review followed the same process as the international experts and national MDA committee. Finally, each provincial group developed specific recommendations for improving the MDA process and care provided.

### Sample, data sources and definitions

All maternal deaths reported to the provincial committees in 2010 and 2017 were included. Data used for the review included case abstracts, data collection forms used in provincial death investigations, and the minutes of audit meetings covering all maternal deaths reported from each subnational committee. These data were sourced from medical records and included medical history, physical, laboratory and imaging test results, treatments and drug orders, as appropriate. For the international review, National Reproductive Health Program members translated these data from Khmer into English. Qualitative data on quality of care and MDA came from participants from these subnational areas. Standard definitions were used for all variables. (*17*)

## Data analysis

The international experts extracted and entered data on the variables of interest from the translated case abstracts into Microsoft Excel, then cleaned and presented them as categorical values. Descriptive statistics (n/N,%; or mean, standard deviation) were calculated for each variable for 2010 and 2017 and compared for statistical significance using χ^2^ and Fisher's exact tests as appropriate, using Epi Info 7.0 (2021).

## Results

### Number and characteristics of maternal death cases reviewed

Nationwide in 2010 and 2017, 176 and 59 maternal death cases were reported, respectively, and were reviewed by the national MDA committee and international experts. Compared to 2010, cases in 2017 had lower parity  (2.1 in 2017 versus 3.6 in 2010) and were significantly more likely to have one (90.0% vs 68.2%) and at least four (48.0% vs 27.3%) antenatal care visits, and for childbirth (52.3% vs 28.7%) and place of death (65.9% vs 46.3%) to be in a public hospital. The timing of death was more commonly during antepartum and postpartum periods (**Table 1**).

**Table 1:** Characteristics of reported maternal deaths, 2010 and 2017, Cambodia

Characteristic	2010 (*n* = 176)	2017 (*n* = 59)	*P*
**Age, mean (standard deviation)**	**31.1 (7.5)**	**32.2 (6.5)**	***NS***
**Parity, mean (standard deviation)**	**3.6 (2.5)**	**2.1 (1.7)**	***0.001***
**Antenatal care by skilled provider**			
**At least one contact**	**60 (68.2)**	**45 (90.0)**	***0.004***
**Four or more contacts**	**24 (27.3)**	**24 (48.0)**	***0.014***
**Place of birth**			
**Home, not attended by skilled attendanta**	**55 (40.4)**	**6 (13.6)**	***0.001***
**Home, attended by midwives**	**4 (2.9)**	**0**	***NS***
**Health centre**	**30 (22.1)**	**12 (27.3)**	***NS***
**Public hospital**	**39 (28.7)**	**23 (52.3)**	***0.004***
**Private health facility**	**3 (2.2)**	**2 (4.5)**	***NS***
**During transfer**	**5 (3.7)**	**1 (2.3)**	***NS***
**Place of death**			
**Home, not attended by skilled attendanta**	**29 (21.3)**	**4 (10.3)**	***NS***
**Home after birth with skilled attendant**	**5 (3.7)**	**1 (2.2)**	***NS***
**Health centre**	**14 (10.3)**	**6 (9.1)**	***NS***
**Public hospital**	**63 (46.3)**	**29 (65.9)**	***0.024***
**Private health facility**	**1 (0.7)**	**1 (2.3)**	***NS***
**During transfer**	**24 (17.6)**	**3 (6.8)**	***NS***
**Timing of death**			***0.0001***
**Antepartum**	**40 (23.7)**	**8 (14.3)**	***–***
**Intrapartum**	**3 (1.8)**	**10 (17.9)**	***–***
**Postpartum**	**119 (70.1)**	**37 (66.1)**	***–***
**Post-abortion**	**7 (4.1)**	**1 (1.8)**	***–***

NS: not statistically significant.

Values are *n*(%) unless otherwise indicated.

^a^ Attended by a traditional birth attendant or relative or unattended

### Cause of death

In both 2010 and 2017, 35–39% of deaths were from postpartum haemorrhage and 10–15% were from preeclampsia/eclampsia (**Table 2**). Less common causes included antepartum bleeding, gestational trophoblastic disease, dehydration, cerebrovascular accident, congestive heart failure, pneumonia and ectopic pregnancy. However, a cause of death could not be assigned (“unclassifiable”) based on the available information in 26.7% of deaths in 2010 and 27.1% in 2017. When a maternal death occurred in another province (for example, in a national referral hospital), the hospital where the death occurred reported the death but did not provide clinical information to the MDA committee of the home province. In such situations, provincial MDA committees relied on information provided by the family to assign the cause of death and identify contributing factors.

**Table 2:** Major causes of death of reported maternal deaths, 2010 and 2017, Cambodia

Cause of death	2010 (*n* = 176)	2017 (*n* = 59)	*P*
**Postpartum haemorrhage**	**63 (35.8)**	**23 (39.0)**	***0.72***
**Preeclampsia/eclampsia**	**27 (15.3)**	**6 (10.2)**	***0.30***
**Abortion-related**	**8 (4.5)**	**1 (1.7)**	***0.32***
**Obstructed labour/ruptured uterus**	**4 (2.3)**	**3 (5.1)**	***0.28***
**Sepsis**	**4 (2.3)**	**2 (3.4)**	***0.64***
**Unclassifiable**	**47 (26.7)**	**16 (27.1)**	***0.95***
**Other**	**23 (13.1)**	**8 (13.6)**	***–***

Values are *n*(%) unless otherwise indicated.

### Contributing factors

The most common contributing factors in 2010 were anaemia (11, 6.3%) and HIV (4, 2.2%). In 2017, anaemia was a contributing factor for two (3.4%) deaths, while HIV was a contributing factor for none. The most common harmful traditional factor noted in 2010 was “roasting” (16, 9.1%), where women in the postnatal period are put in a room with a hot fire for up to a month. No harmful traditional factors were noted in 2017. The number of women who reportedly had an abortion in 2010 and 2017 were eight (4.5%) and one (1.7%), respectively.

### Quality of care

Of the 63 women who died of PPH in 2010, 46 (73.0%) received a prophylactic uterotonic in the third stage of labour versus 22 (95.7%) of the 23 women who died of PPH in 2017 (χ^2^ = 5.2, *P* < 0.02). The use of additional uterotonics was recorded in seven (30.4%) of 23 reports reviewed in 2017 and eight (12.7%) of the 63 reports with available data in 2010 (χ^2^ = 2.1, *P* = 0.14). Delays in the use of additional doses of oxytocin and correction of hypovolemic shock were additionally noted to have contributed to PPH-related deaths in hospitals (**Table 3**).

**Table 3:** Use of medications in the management of women who died of postpartum haemorrhage and preeclampsia/eclampsia from reported maternal death cases, 2010 and 2017, Cambodia

Cause of death and treatment received	2010	2017	*P*
**Postpartum haemorrhage cases**	**63**	**23**	***–***
**Postpartum haemorrhage cases that received:**			
**Oxytocin, prophylactic**	**46 (73.0)**	**22 (95.7)**	**0.02**
**Oxytocin, second dose**	**8 (12.7)**	**7 (30.4)**	**0.14**
**Preeclampsia cases**	**27**	**6**	**–**
**Preeclampsia cases that received:**			
**Magnesium sulfate**	**10 (37.0)**	**4 (66.7)**	**0.18**
**Antihypertensive**	**1 (6.7)**	**1 (16.7)**	**0.48**

Values are *n*(%) unless otherwise indicated.

Among the 27 women who died from preeclampsia/eclampsia in 2010, 10 (37.0%) received magnesium sulfate, but one (3.7%) received it late and in inappropriate doses. Among the six hypertension-related maternal deaths in 2017, four (66.7%) received magnesium sulfate, but one (16.7%) received it late. The use of antihypertensive medication in the management of eclampsia was recorded in one (16.7%) of six reports reviewed in 2017 and one (6.7%) of 15 with available data in 2010 (χ^2^ = 0.50, *P* = 0.48; **Table 3**).

MDA reports in 2010 and 2017 also described disrespectful behaviour by care providers such as shouting and disregarding the women’s complaints. Additionally, during the 2018 review, participants reported that ambulances were stationed at provincial hospitals and were summoned to health centres for emergency transfers. This could have contributed to delays in critical therapeutic management. Moreover, some medicines (for example, oxytocin and magnesium sulfate) or materials in emergency care kits were not replenished in a timely manner. Even when available, they may not have been used as staff do not routinely accompany sick mothers during referral for emergency care or communicate with the receiving hospital.

### Data quality

Information was insufficient to assign the cause of maternal death in over one quarter of cases reviewed in both time periods. Other indicators were also missing data (**Table 4**). Statistical significance was probably limited by the relatively small number of total cases, especially for preeclampsia.

**Table 4:** Missing data per variable in reported maternal deaths, 2010 and 2017, Cambodia

Variable	Missing observations	
	2010 (*n* = 176)	2017 (*n* = 59)
**Age**	**2 (1.1)**	**0 (0.0)**
**Parity**	**9 (4.1)**	**5 (8.5)**
**Place of birth**	**40 (22.7)**	**15 (25.4)**
**Death^a^**		
**Timing**	**7 (4.6)**	**3 (5.1)**
**Place**	**2 (1.1)**	**1 (1.7)**
**Classifiable cause**	**47 (26.7)**	**16 (27.1)**
**Status of baby**	**39 (22.2)**	**37 (72.5)a**

Values are *n*(%) unless otherwise indicated.

^a^ *n* = 51 in 2017, as eight mothers died before delivery of the baby.

### Quality of the MDA recommendations

The expert panel judged that only five (2.8%) MDA recommendations in 2010 and five (42%) of the randomly selected sample of 12 recommendations from 2017 specifically addressed correcting the point along the causal pathway that resulted in maternal death. Most MDA recommendations reviewed in 2010 were vague. Examples include: “arrange and strengthen the referral system of Operational District”; “Health centres should have free referral system for pregnant woman”; and “strengthen management and quality of services.” During the discussion in 2018, participants reported that more specific recommendations related to contributing factors were made during the MDA committee meetings but were not reflected in written recommendations.

### Underreported deaths

Assuming a birth cohort of 360 000, the 176 deaths in 2010 corresponded to a maternal mortality ratio (MMR) of 49/100 000 live births. The Cambodian Demographic and Health Survey (*7*) estimated an MMR of 206/100 000 live births (95% confidence interval [CI]: 124–288), an underreporting of 567 cases (95% CI: 271–863). The United Nations (WHO, the United Nations Children’s Fund, the United Nations Population Fund and the World Bank Group) estimated MMR in 2017 to be 160 (95% CI: 116–221), equivalent to 590 deaths. (*18*) The 59 deaths reported underreports the total deaths by 531.

### Feedback from peer reviews and international experts

The peer review groups arrived at similar conclusions as the international experts and the national MDA committee on the quality of recorded data and recommendations made, and the improvements needed to strengthen MDA implementation.

The international experts who participated in both workshops noted that many participants in 2011 appeared unengaged in the evaluation process. One participant remarked: “If I only knew someone would read my report, I would have written a better-quality report.” In 2018, participants actively assessed the MDA cases and acknowledged gaps in the MDA committee process and recommendations: “This workshop made me realize that there are a lot of mistakes, incomplete and misinformation that could not be analysed in the MDA. Our MDA recommendations are very vague and not specific.”

Observations about care quality included: “health facilities often did not follow the Safe Motherhood Protocol”; “...(sometimes) did not correctly diagnose maternal conditions”; and “…often were late in providing (or did not provide) appropriate lifesaving management and referral.”

The peer review groups recommended that mechanisms should ensure that clinical information about deaths that occurred after referral are available for provincial MDA committee audit meetings, and that records and information are complete. Recommendations should focus on addressing the causal pathway and committees should systematically follow up on the implementation of recommended actions. They further recommended retraining on the management of common conditions (for example, PPH) and life-saving interventions (for example, active management of the third stage of labour and stabilization before referral).

## Discussion

As one of only seven countries to have met the MDG5A target, Cambodia has made remarkable progress towards improving services and survival for women of reproductive age. Our observations of MDA implementation in Cambodia in 2010 and 2017 provide insights into some concrete contributions to the continued improvement in maternal survival, including during public health emergencies.

Compared to 2010, significantly more births and deaths in 2017 occurred in health-care facilities than at home (**Table 1**). The median parity among the deceased decreased from 3 to 2 over the same period. These findings align with the Cambodian Demographic and Health 2010 and 2014 surveys, where the number of home births decreased by 62% (from 45% to 17%) and the total fertility rate decreased from 3.0 to 2.7. (*7*, *8*)

In 2007, the Government of Cambodia offered an incentive to midwife teams for every live birth occurring at health facilities that resulted in a live mother and baby. (*19*) This resulted in a vast national increase in births at health facilities that is recognized globally. (*20*) The Ministry of Health invested nationwide to ensure that a wide range of family planning modalities were available at all health centres and midwives had the capacity to provide them. This is despite Cambodia ranking 159th among 196 countries globally in per capita gross national income. (*21*)

PPH and preeclampsia/eclampsia were the top two causes of maternal death in 2010 and 2017. However, compared to women who suffered maternal deaths in 2010, the women who died in 2017 were more likely to have received prophylactic uterotonics and magnesium sulfate. Among women who died following PPH, those who did not receive active management during the third stage of labour decreased from 27% to 4.3% and subsequent addition of oxytocin from 84% to 70%, though the latter was not statistically significant. Among those who died due to preeclampsia/eclampsia, the percentage of those who did not receive magnesium sulfate or antihypertensives decreased by half, but neither reached statistical significance, presumably due to the small sample size.

Additional interventions besides prophylactic uterotonics and magnesium sulfate are needed to further reduce maternal deaths due to PPH and preeclampsia/eclampsia. When PPH occurs, health-care providers need to recognize the problem early and take timely actions such as adequate fluid infusions, additional uterotonics, tranexamic acid, uterine compression, surgical interventions and blood transfusions. (*22*) Likewise, women with severe preeclampsia/eclampsia should receive antihypertensive medication and adequate fluid management while steps are taken for early termination of pregnancy. (*23*, *24*) Our study focused on additional doses of oxytocin and the addition of antihypertensives for PPH and preeclampsia, respectively. Both interventions need improvement. Delays in referral because of the late arrival of ambulances, use of ambulances without functional emergency kits and lack of staff accompanying women during referrals are additional challenges. These findings are similar to other evaluations that have been conducted. (*25*)

Finally, community interventions for iron, folic acid and calcium supplementation, and aspirin in selected pregnancies and populations could reduce anaemia and preeclampsia and thereby reduce complications immediately before, during and after childbirth. (*26*)

Relevant and specific MDA recommendations increased from 3% among the death reports reviewed in 2010 to 42% in 2017. While many recommendations are more relevant, effective use of data to guide recommendations can be greatly improved.

Compared to 2010, maternal death cases in 2017 had decreased by two thirds. However, these represent only one tenth of the estimated maternal deaths in Cambodia, and efforts should be taken to improve maternal death reporting. Routine health information systems underreport maternal deaths, while surveillance systems may or may not improve accurate counting of maternal deaths. Underreporting of maternal deaths is common and has been reported from Asia and Africa. (*27*-*31*) Underreporting should be suspected when reported deaths are significantly below estimated deaths. One must note that estimates of maternal mortality have wide ranges of uncertainty and are based on events that occurred years earlier, not in real time as with surveillance data. (*32*) More importantly, ensuring corrective actions in response to the findings of even a small proportion of deaths could contribute to improvements in maternal health and survival. (*33*)

The two internationally facilitated reviews used death summary reports submitted by provincial MDA committees in Khmer and translated into English. Accuracy of the contents of these summaries could not be verified. The participants noted that better recording of clinical information would make it easier to understand why the women died and to ensure correct classification of cause of death and appropriate recommendations. Over one quarter of deaths were unclassifiable based on the audit reports. When a maternal death occurred, all relevant information related to care at the different health-care facilities should have been shared with the provincial MDA committees. Timely information sharing between the different levels will contribute to better quality reviews, identification of modifiable factors that lead to maternal deaths and recommended interventions to address them.

Globally, data systems often do not undergo rigorous review. Decision-makers then either base decisions on unvalidated information or ignore the data. This becomes a burden without benefit. Furthermore, improving data quality requires feedback mechanisms to the reporting units. MDSR systems are no exception. This was exemplified in the first workshop, at which the reporter noted that if he had known someone was going to look at the report, he would have done a better job. The participant obviously was doing this for compliance and not quality improvement. In the second workshop, perhaps because the participants had been through this evaluation previously and had an active national MDA committee, with quarterly reviews headed by a Secretary of State, the participants participated more actively and questioned each other. They recognized the need to continue to collect information more precisely and to make more relevant MDA recommendations.

The richness of these evaluation findings shows the value for countries to engage end-users in routinely evaluating information data systems including MDSR. Likewise, it shows the vastly improved maternal care provided in Cambodia between 2010 and 2017. Lastly, it helped identify the actions needed to continue improving routine maternal health services, which is the first step to making them robust and resilient to events that could disrupt service delivery. MDSR needs to be considered as an adjunct to strengthening maternal health-service delivery systems to meet the needs of pregnant women before, during and after public health emergencies.
